# The interpretable machine learning model associated with metal mixtures to identify hypertension via EMR mining method

**DOI:** 10.1111/jch.14768

**Published:** 2024-01-12

**Authors:** Site Xu, Mu Sun

**Affiliations:** ^1^ Ruijin Hospital Shanghai Jiaotong University School of Medicine Shanghai China

**Keywords:** EMR, hypertension, machine learning, metal mixture, NHANES

## Abstract

There are limited data available regarding the connection between hypertension and heavy metal exposure. The authors intend to establish an interpretable machine learning (ML) model with high efficiency and robustness that identifies hypertension based on heavy metal exposure. Our datasets were obtained from the US National Health and Nutrition Examination Survey (NHANES, 2013–2020.3). The authors developed 5 ML models for hypertension identification by heavy metal exposure, and tested them by 10 discrimination characteristics. Further, the authors chose the optimally performing model after parameter adjustment by Genetic Algorithm (GA) for identification. Finally, in order to visualize the model's ability to make decisions, the authors used SHapley Additive exPlanation (SHAP) and Local Interpretable Model‐Agnostic Explanations (LIME) algorithm to illustrate the features. The study included 19 368 participants in total. A best‐performing eXtreme Gradient Boosting (XGB) with GA for hypertension identification by 16 heavy metals was selected (AUC: 0.774; 95% CI: 0.772–0.776; accuracy: 87.7%). According to SHAP values, Barium (0.02), Cadmium (0.017), Lead (0.017), Antimony (0.008), Tin (0.007), Manganese (0.006), Thallium (0.004), Tungsten (0.004) in urine, and Lead (0.048), Mercury (0.035), Selenium (0.05), Manganese (0.007) in blood positively influenced the model, while Cadmium (−0.001) in urine negatively influenced the model. Study participants' hypertension associated with heavy metal exposure was identified by an efficient, robust, and interpretable GA‐XGB model with SHAP and LIME. Barium, Cadmium, Lead, Antimony, Tin, Manganese, Thallium, Tungsten in urine, and Lead, Mercury, Selenium, Manganese in blood are positively correlated with hypertension, while Cadmium in blood is negatively correlated with hypertension.

## 1 INTRODUCTION

The quantity of individuals with hypertension has multiplied since 1990, and 1.28 billion adults worldwide have hypertension currently.^1^ Risk factors for hypertension include obesity, lack of physical exercise, alcohol consumption, etc.^2^ According to epidemiological data, exposure to environmental metals is associated with hypertension.^3^

Genetics, diet, and lifestyle are currently well‐established risk factors for hypertension,^4^ while previous researches have suggested that metal exposure may also be the cause of hypertension's etiology.^5^ Metals can get into people's bodies in a variety of ways, such as air inhalation, skin contact, and digestion.^6^ Essential elements could assume an imperative part in human physiological activities, including immunity, metabolism, and development.^7^ However, insufficient, or excessive essential elements might apply antagonistic impacts on people health.^7^, ^8^ The harmful metals can disrupt the body's homeostasis and harm organs.^9^ Numerous epidemiological studies have focused on metal exposure's impacts on hypertension, however the results are still uncertain. For example, the positive correlation was found in a systematic review between blood cadmium levels and hypertension prevalence in adults,^10^ but a cross sectional study in Canada^5^ showed that urinary cadmium was inversely correlated with blood pressure among the general adults. The conflict between research results might be due to study designs, or exposure levels, therefore further studies are necessary to confirm the association.

People health, including hypertension occurrence, is often influenced by metal mixtures with joint impact,^11^ however, most studies have only concentrated on specific metal exposure,^5^, ^9^, ^12^, ^13^, ^14^ using traditional statistical or ML analysis.^15^, ^16^, ^17^ In this way, a new analytical approach could be used to better identify the association between hypertension and heavy metal exposure.

In the methodologies traditionally used to identify diseases, there are numerous required standards for preparing datasets.^12^, ^13^, ^14^ With developments of computer science and expanding information sources, researchers have faced a huge challenge when mining hidden meanings from big data.^18^ Due to the black‐box nature, ML requires less standards for preprocessing data, which increases the possibility to analyze numerous information, which can support hazard identification, or other decision‐making for health.^19^

In our study, we chose NHANES (2013−2020.3) datasets for mining the connection between hypertension and heavy metal exposure. We selected 5 ML models to identify hypertension by heavy metals' exposure, compared the performance characteristics of the 5, and then used Genetic Algorithm (GA) to improve the efficiency of the best. Further, the study incorporated the advanced EMR mining technique based on SHAP^20^ and Local Interpretable Model‐Agnostic Explanations (LIME)^21^ into the evaluation of heavy metals’ contribution during the identification of hypertension, boosting the likelihood of early intervention.

## 2 METHODS

### 2.1 Participants

Through various survey strategies, US NHANES study investigated the US population for demographics, dietary, examination, laboratory, and questionnaire data. All data can be found on the website of the American Centers for Disease Control and Prevention (https://www.cdc.gov/nchs/nhanes). The sample of our study consisted of three contiguous cycles from 2013 to March 2020 of NHANES.

The data selected for the study included the following inclusion criteria: participants were over 20 years old; participants had participated in blood and urine tests of heavy metals; and NHANES questionnaire data contained participants' hypertension status information. Exclusion criteria included: individuals with more than 10% missing values were dropped; individuals with any contradictory information were dropped. Finally, the follow‐up analysis included a total of 19 368 participants.

### 2.2 Data collection

#### 2.2.1 Demographics characteristics of the study participants

Participants' demographics and other relevant characteristics were collected in NHANES. Characteristics included gender, age (in years at screening), Race/Hispanic origin w/ NH Asian, education level (college or above, high school or equivalent, and less than high school), poverty‐to‐income ratio (PIR) (≤1, 1−4, and ≥4),^22^ and body mass index (BMI, kg/m^2^).

#### 2.2.2 Heavy metals

Our analyses included urinary and blood level of 16 heavy metals. At the National Center for Environmental Health, rigorous quality steps were used to detect all heavy metal concentrations.^23^

#### 2.2.3 Outcome ascertainment

Since the 2013−2014 data release cycle, professional physicians have used the code I10, which was labeled in accordance with the International Statistical Classification of Diseases and Related Health Problems, Tenth Revision (ICD‐10), to diagnose hypertension in NHANES.^24^

### 2.3 Pre‐processing of features

In our research, we selected 22 variables (also known as features in ML field). Among them, 19 were continuous, and 3 were categorical. We eliminated data with loss rate of 10% and above. Continuous variables had their median filled in for missing values, whereas unordered categorical variables had their mode filled in, ordinal categorical variables had their nearest neighbor values filled in. We used Standard Scaler for standardizing features and one‐hot codes for transforming categorical variables in the ML model.^25^ We used Principal Component Analysis (PCA) and the Select K Best (SKB) algorithm for extracting features.^26^ We removed variables that have little contribution to the model during preprocessing in order to avoid overfitting.

### 2.4 Model establishment

Data of study was split into train set and test set by repeated K‐Fold cross validation.^27^ We employed 5 ML algorithms, including Neural Networks (DNN), Support Vector Machine (SVM), Gaussian Naive Bayes (GNB), Decision Tree (DT), and eXtreme Gradient Boosting (XGB), to establish models for the identification of hypertension by heavy metal exposure. These five models have their own characteristics. The DNN method is usually more accurate with simple structure for data training; meanwhile, it also has strong black‐box characteristics, that is, it is more difficult for people to understand its discrimination principle.^28^ SVM is data‐insensitive, but can process nonlinear, multidimensional datasets.^29^ GNB performs well on small‐scale data, can handle multiple classification tasks, and is suitable for incremental training, but there will be noise and redundancy.^30^, ^31^ Visual analytics are supported by DT, which is easy to comprehend and interpret, but it is susceptible to problems with over‐fitting.^32^ XGB is a library optimized to increase distributed gradient and designed to be highly efficient, flexible, and portable^33^; however, XGB's model parameters are too many to adjust for the optimal efficiency.^34^

We chose the model that was best suited for identifying diseases after comparing the discrimination features of the five models, and then used GA to adjust parameters in solving disadvantage of the chosen. The SHAP and LIME value were used to illustrate our model with related risk variables for hypertension identification in participants from 2013 to March 2020.^35^ SHAP was used for overall interpretation, and LIME was used for partial interpretation.

### 2.5 Statistical analysis

The continuous variable was described as the median (interquartile range), and the categorical variable was described as the number (percentage). The chi‐square test was used to compare various group‐specific characteristics. Geometric means (geometric standard deviations) were used to describe heavy metals. Throughout the 8+ years (three data release cycles), the trends were examined using the Mann‐Kendall test.

The indicators used for model effectiveness testing included average area under the curve (AAUC)^36^ and 95% confidence intervals (95% CI), best AUC (BAUC), average precision score (APS), average recall, average f1 score, average accuracy, average brier score loss, average cross‐entropy loss, average Jaccard index, and average Cohen's kappa of each model by repeated K‐Fold cross validation.

**FIGURE 1:**
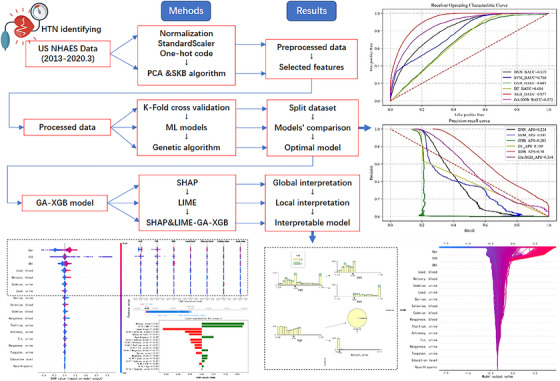
Overview plot.

Python 3.9.7 was used for all analyses, with *p* < .05 considered statistically significant. An overview of our methodology is shown in Figure 1.

## 3 RESULTS

### 3.1 Participants’ demographics characteristics

The characteristics of study participants are summarized in Table 1. We analyzed 19 368 participants in total. Among them, 2650 were diagnosed as hypertension, 9397 were men, and the average age was 57. Hypertension participants were more likely to be older, have the higher level of BMI, non‐Hispanic white, and have the education level of college or above (all *p* < .05).

**TABLE 1:** The study participants’ characteristics in NHANES (2013−2020.3).

Characteristics	Total (*n* = 19368)	With HTN (*n* = 2650)	Without HTN (*n* = 16718)	*p*
Gender, %				.989
Men	9397 (48.52)	1299 (49.02)	8098 (48.44)	
Age, *y*	57 (33‐69)	64 (56‐72)	54 (28‐68)	<.001^*^
BMI, kg/m^2^	28.4 (23.8, 33.6)	30.5 (26.7, 35.4)	28.2 (23.3, 33.2)	<.001^*^
Race/Hispanic, %				<.001^*^
Mexican American	2605 (13.45)	264 (9.96)	2341 (14)	
Other Hispanic	1888 (9.75)	230 (8.68)	1658 (9.92)	
Non‐Hispanic White	7777 (40.15)	977 (36.87)	6800 (40.67)	
Non‐Hispanic Black	4502 (23.24)	864 (32.6)	3638 (21.76)	
Non‐Hispanic Asian	1673 (8.64)	236 (8.91)	1437 (8.6)	
Other Race	923 (4.77)	79 (2.98)	844 (5.05)	
Education level, %				<.001^*^
College or above	11603 (59.91)	1306 (49.28)	10297 (61.59)	
High school or equivalent	4027 (20.79)	720 (27.17)	3307 (19.78)	
Less than high school	3738 (19.3)	624 (23.55)	3114 (18.63)	
PIR, %				.245
High	3826 (19.75)	558 (21.06)	3268 (19.55)	
Medium	11368 (58.69)	1571 (59.28)	9797 (58.6)	
Low	4174 (21.55)	521 (19.66)	3653 (21.85)	

Abbreviations: BMI, body mass index; PIR, poverty to income ratio.

* *p* < .05.

### 3.2 Heavy metals’ concentrations

The heavy metal concentration in urine or in blood of each data release cycle are described in Table 2. Based on the data release cycles, Barium, Cadmium, Cobalt, Cesium, Manganese, Lead, Antimony, Tin, Thallium, and Tungsten in urine, and Lead, Cadmium, Mercury, Selenium, and Manganese in blood showed significant tendencies (all P_for trend_ < 0.05).

**TABLE 2:** Mean values of heavy metal concentration by each NHANES (2013−2020.3) data release cycle.

	Cycles of NHANES			
Heavy metal	2013−2014	2015−2016	2017−2020.3	P_for trend_
**In urine**				
Barium (ug/L)	1.6 (2.68)	1.59 (2.25)	1.45 (2.34)	<0.001^*^
Cadmium (ug/L)	0.34 (0.44)	0.32 (0.39)	0.39 (0.55)	<0.001^*^
Cobalt (ug/L)	0.68 (2.09)	0.66 (1.52)	0.56 (1.12)	<0.001^*^
Cesium (ug/L)	4.86 (3.24)	4.96 (3.83)	5.11 (3.47)	<0.001^*^
Manganese (ug/L)	51.45 (49.43)	54.5 (50.45)	51.12 (49.21)	<0.001^*^
Molybdenum (ug/L)	0.19 (0.76)	0.15 (0.42)	0.16 (0.3)	0.424
Lead, urine (ug/L)	0.49 (0.7)	0.48 (0.62)	0.45 (0.55)	<0.001^*^
Antimony, urine (ug/L)	0.07 (0.12)	0.08 (0.28)	0.07 (0.15)	<0.001^*^
Tin, urine (ug/L)	1.89 (6.21)	1.49 (3.08)	1.46 (3.63)	<0.001^*^
Thallium, urine (ug/L)	0.17 (0.14)	0.18 (0.16)	0.19 (0.14)	<0.001^*^
Tungsten, urine (ug/L)	0.14 (0.46)	0.13 (0.26)	0.12 (0.4)	<0.001^*^
**In blood**				
Lead (ug/dL)	1.33 (1.57)	1.24 (1.12)	1.18 (1.05)	<0.001^*^
Cadmium (ug/L)	0.46 (0.52)	0.43 (0.5)	0.48 (0.65)	<0.001^*^
Mercury, total (ug/L)	1.37 (2.46)	1.22 (2.0)	1.17 (2.28)	<0.001^*^
Selenium (ug/L)	194.46 (26.82)	189.79 (25.5)	183.93 (26.83)	<0.001^*^
Manganese (ug/L)	9.78 (3.72)	10.17 (3.71)	9.58 (3.69)	<0.001^*^

* P < 0.05.

### 3.3 Models’ preprocessing

In feature selection, PCA determined that at least 18 variables were required to retain more than 90% content of the original information, and SKB scores of features ranged from 0.01 to 1083.44. We selected the top 18 features by scores to adapt our ML models, then 5 ML algorithms were applied to NHANES datasets using repeated K‐Fold cross validation to train the models.

### 3.4 Models' performance

**FIGURE 2:**
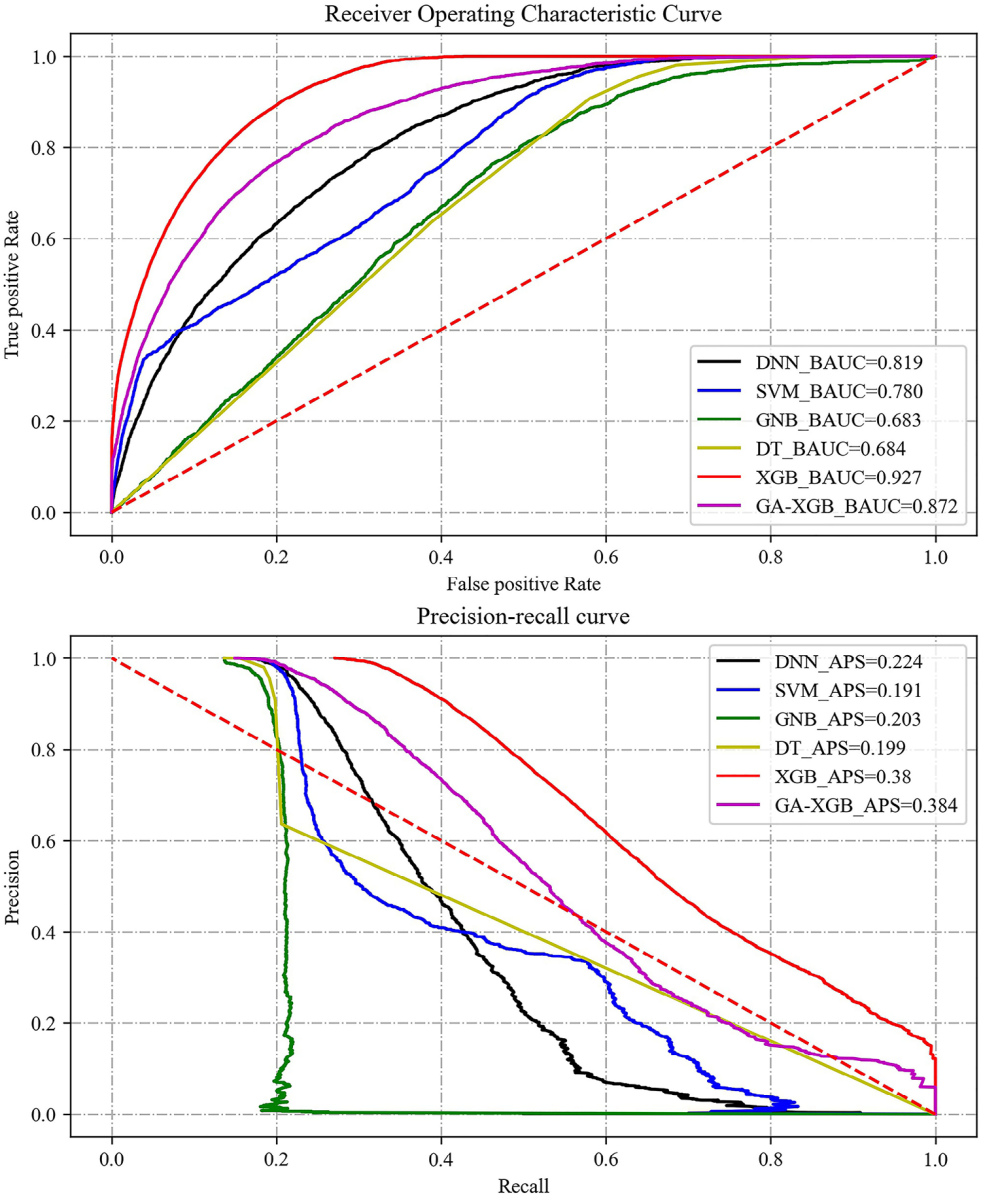
The best receiver operating characteristic curve and precision‐recall curve for models.

The XGB model has the optimal AAUC (AUC: 0.766; 95%CI: 0.763−0.769), BAUC (0.927), and APS (0.38) performance which were significantly higher than the AUC values of the other four models (*p* < .05). In order to pursue better AAUC and APS in identifying hypertension, we adopted GA for parameter adjustment and obtained GA‐XGB model with the best performance of AAUC (AUC: 0.774; 95%CI: 0.772−0.776), and APS (0.384). The best receiver operating characteristic (ROC) curve and precision‐recall curve of 6 ML models (including GA‐XGB) are shown in Figure 2. DNN (85.7%), SVM (86.3%), DT (86.3%), XGB (85.8%), and GA‐XGB (87.7%) showed good accuracy when identifying hypertension.

### 3.5 Models’ comparison

Table 3 shows the performances' comparison of the ML models. The AAUC, BAUC, APS, average recall, average f1 score, average accuracy, average brier score loss, average cross‐entropy loss, average Jaccard index, and average Cohen's kappa for all 5 ML models are shown in Table 3. XGB reached the best of the five models in 5 of the 10 performance indicators. Typically, the AAUC (AUC: 0.766; 95%CI: 0.763−0.769), BAUC (0.927), and APS (0.38) of XGB performed the best of all 5 ML models. The comparison results demonstrate that XGB has the best performance of the five for hypertension identification. Then, we used GA to adjust the parameters of XGB to further improve its model effect, as shown on the far right of Table 3.

**TABLE 3:** Comparison of ML models’ performance.

Characteristics	DNN	SVM	GNB	DT	XGB	GA‐XGB
AAUC	0.698 (0.695, 0.702)	0.647 (0.644, 0.65)	0.678 (0.674, 0.682)	0.678 (0.675, 0.681)	0.766 (0.763, 0.769)	0.774 (0.772, 0.776)
BAUC	0.819	0.78	0.683	0.684	0.927	0.872
APS	0.224	0.191	0.203	0.199	0.38	0.384
Average recall	0.024	0	0.495	0	0.194	0.102
Average f1 score	0.044	0	0.291	0	0.272	0.184
Average accuracy	0.857	0.863	0.677	0.863	0.858	0.877
Average brier score loss	0.115	0.118	0.198	0.111	0.11	NA
Average cross‐entropy loss	0.374	0.398	0.772	0.361	0.367	4.123
Average Jaccard index	0.023	0	0.17	0	0.159	0.101
Average Cohen's kappa	0.071	0	0.128	0	0.416	0.163

Abbreviations: AAUC, average area under the curve; APS, average precision score; BAUC, best area under the curve; DNN, deep neural networks; DT, decision tree classifier; GNB, Gaussian naive bayes; NA, null; SVM, support vector machine; XGB, extreme gradient boosting;

### 3.6 Feature importance visualization

**FIGURE 3:**
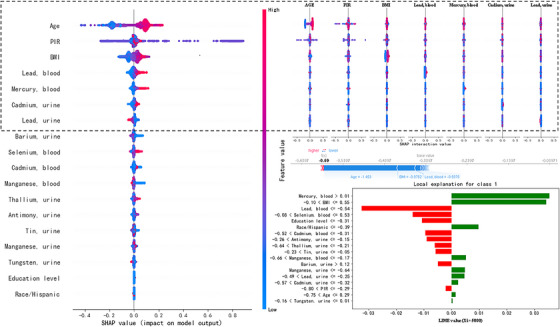
The SHAP&LIME‐GA‐XGB summary plot.

SHAP and LIME were used to visualized features' influence on hypertension identification of the GA‐XGB model. The SHAP&LIME summary plot demonstrates the impact of each selected feature of the model to identify hypertension (Figure 3).

The SHAP value plot on the left side of Figure 3 globally indicates that Barium (0.02), Cadmium (0.017), Lead (0.017), Antimony (0.008), Tin (0.007), Manganese (0.006), Thallium (0.004), Tungsten (0.004) in urine, and Lead (0.048), Mercury (0.035), Selenium (0.05), Manganese (0.007) in blood positively influence the model, while Cadmium (−0.001) in urine negatively influence the model. Additionally, the SHAP&LIME summary plot shows that being old, non‐Hispanic, having a lower education level, having a higher PIR, and having a higher BMI are related to higher hypertension risk. The SHAP interaction value plot on the upper right side of Figure 3 demonstrates the interaction between main features. The LIME value plot on the lower right side of Figure 3 locally indicates the feature importance of single sample discrimination (the 5000^th^ sample). SHAP values illustrate features’ contributions to hypertension identification of the model.

### 3.7 Prediction interpretation

**FIGURE 4:**
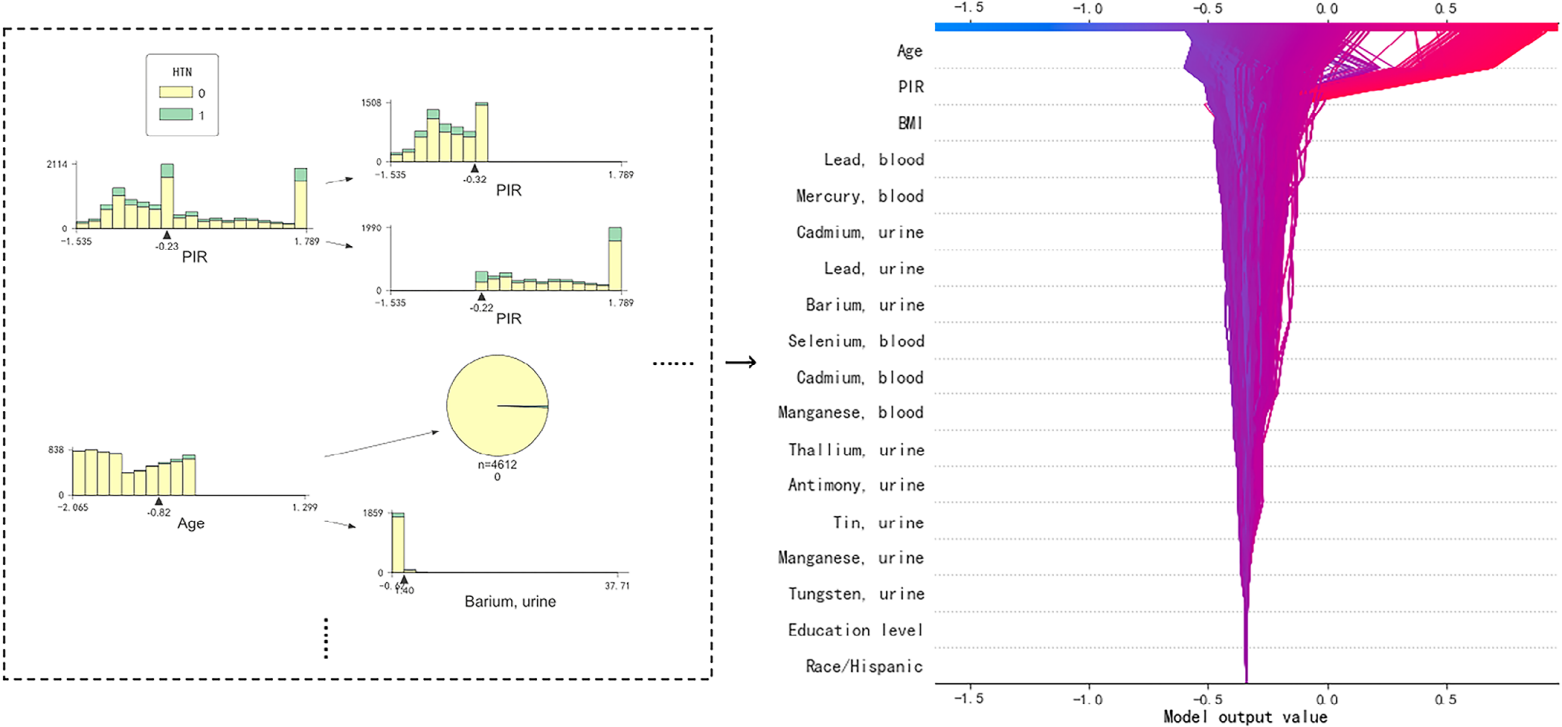
The SHAP‐GA‐XGB decision plot.

In the SHAP decision plot on the right side of Figure 4, each participant is represented by each line. The lines converge to the single point of 0.877. These features are listed in descending order based on the plotted observations. The tree plot on the left side of Figure 4 shows the optimal logic of discrimination, and served as one of basic trees of the decision logic.

## 4 DISCUSSION

In our study, for identifying hypertension in 2013−2020.3 NHANES data, we developed a ML strategy that can be understood in relation to heavy metal exposure. The GA‐XGB model was chosen to identify hypertension because it performed the best of the 5 ML algorithms. The GA‐XGB model performed well with an average AUC of 0.774, and an accuracy of 0.877. Meanwhile, using the SHAP game theory method with LIME could make up for the shortcomings of these two algorithms, and demonstrate the significance of each model feature both globally and locally, with the summary and decision plot. Our results indicated that the SHAP&LIME‐GA‐XGB model had encouraging possibility for hypertension identification by heavy metal exposure.

Our research is based on earlier studies that used ML algorithms to predict diseases [27, 33, 35]. They discovered that sophisticated categorization algorithms can increase prediction accuracy.

Mathematical algorithms are used in ML, a subset of artificial intelligence, to find and classify structures in heterogeneous data so that decisions can be made.^18^, ^37^ It is difficult to understand whether certain conclusions will be drawn when considering the ML algorithm.^38^ In the meantime, medical decision‐making has been hindered by ML algorithms' inability to be understood.

There are several notable characteristics of our SHAP&LIME‐GA‐XGB model. First, it used multi‐source NHANES's demographics, examination, laboratory, and questionnaire data for applying to ML models rather than building a new data survey or collection. Additionally, the United States placed a significant emphasis on heavy metal exposure through a variety of environment programs beginning in 2013.^39^ Also from 2013, the standard ICD‐10 of hypertension has been applied to disease records of NHANES. Environmental heavy metal exposure levels decreased directly as a result of policy and treatment programs, while hypertension incidences also varied.^40^ We utilized enormous amounts of data to develop ML models, focusing on the heavy metal concentration in each participant's urine and blood. The AAUC of the GA‐XGB model was 0.774, demonstrating good efficiency. In addition, we used 5 ML algorithms, which have been described in other ML studies to address hypertension^12^, ^13^, ^14^ or other diseases, to identify hypertension by heavy metal exposure. Some of them were efficient and applicable to raw data. Particularly, the accuracy of algorithm prediction improved with the improvement of data authenticity.^41^ Further, we evaluated the multi‐level prediction potential of ML models. The GA‐XGB model had the best performance in terms of classification robustness, according to the comprehensive comparison results by 10 discrimination characteristics. At the same time, we effectively avoided the occurrence of over‐fitting or under‐fitting by repeated K‐Fold segmentation. We applied SHAP values for global interpretation and LIME values for local interpretation to the GA‐XGB model with the intention of achieving the best interpretability, because it is hard to correctly comprehend the ML methodology and to visually demonstrate the identification results. A positive SHAP value indicated that there was a positive conditional association between the related feature and hypertension, whereas negative SHAP values implied the opposite. The SHAP with tree‐explainer can visualize the decision‐making process of the model.

The findings of SHAP were comparable to those of previous studies, which primarily focused on determining how heavy metal exposure affects hypertension. The hazard ratios of the highest quartiles Cadmium compared with the reference group was 1.42 (95% confidence interval [CI] 1.09−2.02) for cases of hypertension, which suggested that high levels of Cadmium might increase the incidence of hypertension.^10^ In addition, Systolic blood pressure and diastolic blood pressure were statistically significantly associated with peak blood Lead level. A blood lead level ≥ 6.87 μg/dL was associated with hypertension.^42^ Moreover, heavy metals showed pertinence for special populations. The study suggested that increased Manganese during pregnancy might be a potential risk factor for inducing pregnancy hypertension,^43^ and urinary Antimony was consistently and dose‐responsively associated with increased blood pressure and hypertension, of which Antimony was the major contributor among children.^44^

When it comes to the analysis and explanation of particular features, experts will benefit in the future from constant tracking because it will help them come to logical conclusions rather than simply accepting the algorithm's predictions. Further studies could also focus on validating the model's performance by expanding the database and increasing the participation of clinicians’ judgment.^45^

### 4.1 Limitations

The study has several limitations. First, other characteristics, which might have demonstrated dynamic correlations because of computational constraints when analyzing limited access data, were not disaggregated. Second, although the ICD‐10 is normative, the diagnosis of hypertension was self‐reported by participants in questionnaire data of NHANES,^46^ which may have introduced information bias. The ML models' ability to accurately identify hypertension may have been affected in some way by any incorrect hypertension classification that resulted. Third, numerous data were missing due to the strict including criteria of the study participants, which may has probably led to the bias. Finally, model's complexity of interpretation may limit their reproducibility.

## 5 CONCLUSIONS

In our study among US NHANES 2013−2020.3 participants, the SHAP&LIME‐GA‐XGB model was found to be an interpretable ML model with high efficiency and robustness that identifies hypertension based on heavy metal exposure. Barium, Cadmium, Lead, Antimony, Tin, Manganese, Thallium, Tungsten in urine, and Lead, Mercury, Selenium, Manganese in blood positively contribute to hypertension, while Cadmium in blood negatively contributes to hypertension.

## AUTHOR CONTRIBUTIONS

All authors contributed to designing the study. Site Xu was responsible for data collection and analysis. Site Xu was responsible for writing the manuscript. The corresponding author Sun attested that all listed authors meet authorship criteria. No other individuals meeting the criteria have been omitted. Sun is the guarantor. All authors have read and approved the final manuscript.

## CONFLICT OF INTEREST STATEMENT

The authors declare that they have no competing interests.

## CONSENT FOR PUBLICATION

Informed consent was obtained from all individual participants included in the study.

## Data Availability

The datasets that support the findings of this study are available publicly. Full lists of records identified through database searching are available on reasonable request from the authors.
